# Osteoimmunological Principles Adapted to Achieve Mechanically Superior Posterolateral Fusion in a New Zealand White Rabbit Model Using Antigen-Coated, Electrospun Beta-Tricalcium Phosphate

**DOI:** 10.7759/cureus.62781

**Published:** 2024-06-20

**Authors:** Loubert S Suddaby, Douglas C Fredericks

**Affiliations:** 1 Department of Neurosurgery, Erie County Medical Center, Buffalo, USA; 2 Iowa Spine Research Lab, Department of Orthopaedics and Rehabilitation, University of Iowa, Animal Research Surgicenter, Iowa City, USA

**Keywords:** spinal fusion, bone substitutes, bone morphogenetic protein, beta-tricalcium phosphate, autografting, antigens

## Abstract

Introduction

Triggering the immune system via antigenic stimulation at the time of spinal fusion surgery may enhance bone morphogenesis and result in successful bony arthrodesis. We sought to demonstrate that bone morphogenesis could be enhanced via antigenic immunologic stimulation of a surgical fusion site.

Methods

New Zealand white rabbits underwent non-instrumented posterolateral fusion of L5-6 with implantation of either an immunologically activated graft (inert beta-tricalcium phosphate) or harvested autograft. Fusion was evaluated using plain radiographs, micro-computed tomography (CT), mechanical palpation, and biomechanical testing. The final evaluation was carried out at 12 weeks postoperatively.

Results

Eight rabbits received immunologically activated grafts; 10 received autografts and served as historical controls. Fusion rates were identical between groups (both 50%). Radiographs and micro CT of the fusion mass showed no significant difference between groups, and both showed good incorporation of the transverse processes into the fusion masses with radiographic evidence confirming trabeculation and bone remodeling. However, mechanical testing of the fusion sites showed superior fusion strength in the rabbits that received immunologically activated grafts, approaching a factor of two on flexion/extension, lateral bending, and axial rotation. Little to no graft material was appreciable in the non-fused antigen-treated specimens.

Conclusions

There is a long-standing need for a graft material that can replace autograft bone, due to the negative clinical consequences and financial costs pertaining to autologous bone harvesting. No allograft bone substitute to date has been able to reliably replicate the success of harvested autograft bone. This study suggests that immunological enhancement of inert beta-tricalcium phosphate can potentially be a substitute for allograft bone that can meet and even exceed the success of harvested autograft bone.

## Introduction

In 2002, recombinant human bone morphogenetic protein-2 (rhBMP-2) was approved by the US FDA for use in bone fusion procedures. Unfortunately, safety concerns about rhBMP-2 arose after its widespread adoption, with reported adverse effects including osteolysis, heterotopic bone formation, bone cyst formation, dysphagia, retrograde ejaculation, arachnoiditis, wound seroma formation, and neurologic deficits secondary to the profound inflammatory response stemming from BMP administered at doses 1,000 times higher than found in natural bone healing. The FDA subsequently amended its approval [1-4].

The sheer number of fusion procedures performed annually in the USA alone attests to the importance of bone healing [5,6]. Autologous bone is currently considered the best option for graft material but its adoption is limited by complications including pain, bone site morbidity, and donor site infection. Many surgeons instead opt for less intrusive graft implants that avoid donor site complications and morbidities associated with autologous bone harvesting, but these alternatives have reduced efficacy [7].

A safe and effective option that utilizes inert, calcium-containing substances would be a valuable alternative to autograft bone. The burgeoning field of osteoimmunology holds great promise for such a prospect, although the process of linking the immune system to bone morphogenesis has been in evolutionary practice and development for some 400 million years [8]. Bone morphogenesis occurs via a complex pathway much like the hematologic coagulation cascade, whereby a complex series of biological reactions and interactions lead to blood clot formation. To achieve successful bone formation and fusion, a number of key cellular components as well as a plethora of communicating cytokines are necessary and quantities of these components vary dramatically by individual, body site, and surgical procedure [9]. To be sure, it would be exceedingly difficult to develop an implant that could successfully replicate the features of the bone morphogenetic cascade. It would be presumptuous - perhaps even arrogant - to presume that a solitary cytokine (e.g., BMP) or reconstituted stem cell mixture would facilitate successful bone healing - particularly the complex bone healing required after surgical fusion of the spine.

To date, no graft implant has reliably equaled or exceeded the success rate of autograft bone, which largely contains all cellular components, cytokines, and associated structural elements necessary to facilitate bone healing. Autograft bone has no histocompatibility issues that might trigger an adverse immunologic response, and the amounts and requisite numbers of key components can be tailored such that bone growth and union of spinal segments resulting in a solid living bond occurs.

Here, we used an inert scaffold composed of the main chemical component of bone (calcium phosphate) in an animal model to understand the role of the immune system in facilitating bone morphogenesis during spinal arthrodesis. Using antigenic stimulation, we postulated that inert calcium phosphate might be effective in fusing adjacent spinal elements - perhaps even as effective as transplanted autograft bone. This would require that the bone graft material would be not only osteoconductive and inductive, but also osteopreparatory whereby all integral cellular components would be drawn to the surgical site, enter, and assimilate the calcium phosphate graft, and reconfigure or transmute it to the normal trabeculated bone. This pilot study was designed to ensure that other inducing elements (e.g., stem cells, cytokines, instrumented hardware) would not obfuscate the results so that the value of incorporating the immune system into the fusion process could be judged on its own merits.

## Materials and methods

Animal model

This study was performed in the Animal Research Surgicenter of the Iowa Spine Research Lab at the University of Iowa (IACUC #0041977), in accordance with Institutional Animal Care and Use Committee guidelines. Male New Zealand white rabbits were kept at the animal facilities of the Iowa Carver College of Medicine. The rabbits were maintained in rooms with controlled light, temperature, and humidity, and had access to food and water ad libitum. In total, eight rabbits received immunologically activated grafts, and 10 received harvested autografts.

Preparation of the immunologically activated graft

The antigen was obtained from Sigma-Aldrich. Five micrograms of lipopolysaccharides (LPS) were obtained from Escherichia coli O55:B5 and five micrograms of lipoteichoic acid (LTA) from Staphylococcus aureus. The electrospun beta-tricalcium phosphate, obtained from ORTHOReBIRTH, was divided into two equal pieces (i.e., grafts) and then soaked in a 50/50 combination of both antigens (one L saline containing five micrograms each of LPS and LTA) before being air dried for three hours. Once dried, the grafts were ready for surgical implantation along the decorticated transverse processes (L5-6) of the animal model.

Surgical procedure

After being anesthetized and prepared in a sterile fashion, the New Zealand white rabbits underwent non-instrumented, posterolateral intertransverse surgical fusion at the L5-6 level. The rabbits received either the immunologically activated graft or the harvested autograft product to bridge the decorticated transverse processes. Autograft implants were never exposed to antigenic implants or vice versa. The autograft specimens served as historical controls.

Assessment of fusion quality

Radiographs were obtained immediately postoperatively and again at 8 and 12 weeks; micro-computed tomography (CT) was also carried out at 12 weeks. The size of the fusion mass as well as the incorporation of the transverse processes were assessed. One author (D.C.F.) conducted fusion assessments, via mechanical testing (manual palpation, flexion/extension testing, lateral bending, and axial rotation) of the fusion site and micro CT to assess bony trabeculation of the fusion graft and full incorporation of the transverse processes.

## Results

**Figure 1:**
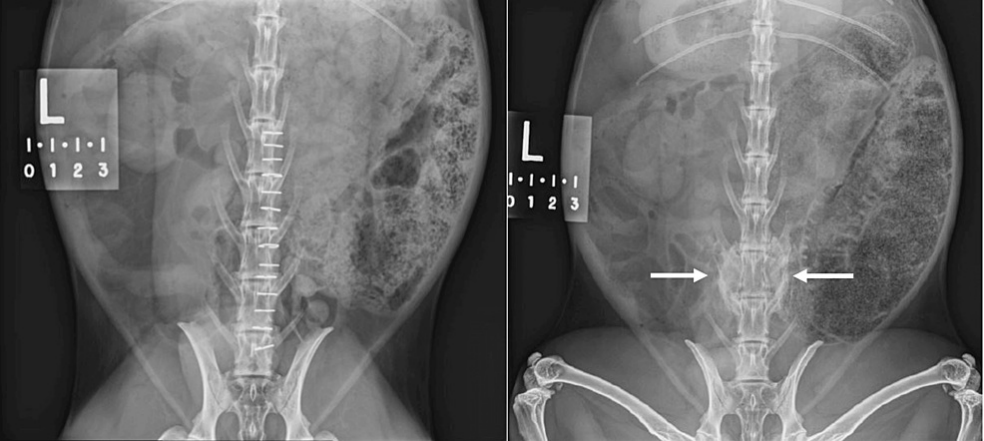
Anteroposterior radiographs of a New Zealand white rabbit depict the fusion of L5-6 with antigenic graft immediately postoperatively (left) and at 12 weeks (arrows, right).

**Figure 2:**
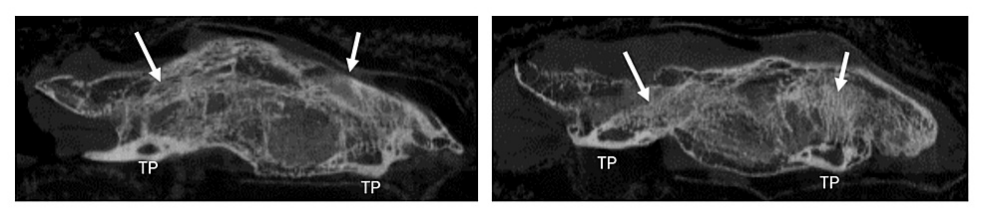
Sagittal micro-computed tomograms of a New Zealand white rabbit depict the fusion of L5-6 with antigenic graft at 12 weeks (arrows). TP: transverse process

As noted, eight rabbits received immunologically activated grafts, and 10 received harvested autografts. All animals that underwent graft implantation survived; no complications were observed and there was no mortality in this study. Good incorporation of transverse processes and trabeculation of graft were seen in rabbits from both groups, and the fusion rate was the same between the immunologically activated graft and autograft groups (both 50%) (Figures 1-2).

**Figure 3:**
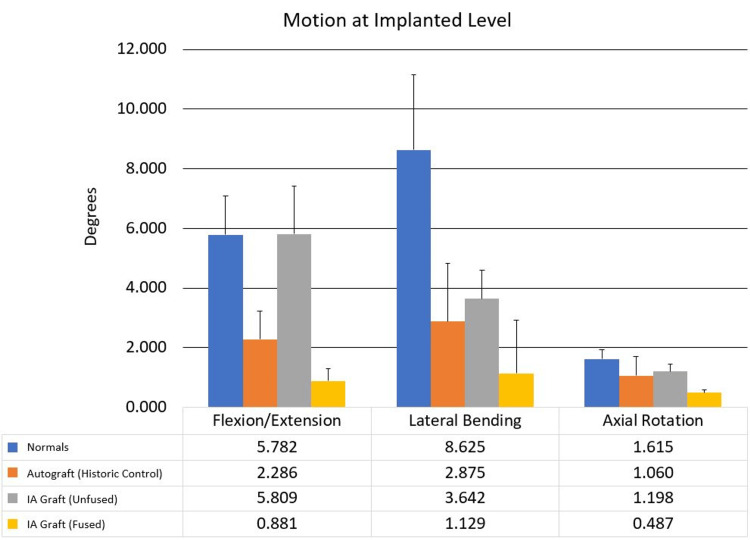
Comparison of mechanical stiffness shows that subjects receiving the immunologically activated graft exceeded the autograft fusion strength by a factor of two. IA: immunologically activated

Although no substantial difference was observed between the grafts in fusion rates, clear differences were observed in fusion when the mechanical stiffness of the fusion mass was tested. The fusion strength in rabbits that received the immunologically activated graft exceeded the autograft fusion strength by a factor of 2 (Figure 3). Because the composition of the immunologically activated graft is primarily radiolucent, ultrasound CT provided sufficient information to determine new bone growth. Micro CT demonstrated normal bone trabeculae.

## Discussion

This pilot study suggests that recruiting the immune system to aid in bone fusion and healing presents a remarkable opportunity to improve on current strategies and techniques for bone fusion and repair. This study further suggests that involving the immune system in surgical fusion procedures (and potentially in the treatment of non-union fractures) is a novel and exciting opportunity whereby even inert calcium phosphate-containing compounds or scaffolds, or implants coated with such substances, can potentially surpass the fusion success of the gold standard autograft.

In the evolution of skeletal organisms, a pathogen-contaminated compound bone fracture would represent a calamitous injury; therefore, it is perhaps no surprise that bone healing and the immune system are cooperatively designed and intertwined to react to this potentially life-threatening dilemma [10]. Bacterial antigens are some of the most potent stimulants of the immune system, so it stands to reason that triggering the immune system via antigenic stimulation coincident with a spinal fusion would enhance bone morphogenesis and result in superior bony arthrodesis [11].

Some of the earliest cellular components in a fracture site are macrophages, either locally situated (i.e., osteomacs) or attracted from the bloodstream in the form of monocytes. Macrophages can be attracted to a traumatic site (including during surgery) through components of cellular debris or through the antigenic components of invading organisms (bacterial antigens). The combination of traumatic debris coupled with antigenic stimulation puts the immune system in a “panic” mode, and circulating monocytes (M0) follow the chemical gradient of antigens, cytokines, and cellular debris components to the site (osteopreparatory). Upon arrival at the traumatic site, M0 monocytes transform into phagocytic macrophages (M1) called classically activated macrophages, consequential to their exposure to antigens, and then begin to remove cellular debris and invading organisms. Local tissue macrophages can be recruited in a similar manner. Circulating monocytes are known to be mutable and have the capacity to change their cell morphology and function into that of tissue macrophages [12]. This association is clearly necessary since a bone fracture requires the presence of a cell that can remove traumatic debris and pathogens to clear the way for the restoration of normal bone morphology and function [13].

In cases of compound fracture, it is imperative that the immune system be involved early since contamination by pathogens invariably occurs. Additionally, studies have shown a direct link between osteoblast differentiation and macrophage polarization, further strengthening the hypothesis that macrophages profoundly influence bone morphogenesis [14]. Macrophages not only promote osteoblast differentiation and maturation, but can also recruit mesenchymal stem cells to an injury site, as well as aid in stimulating adjunctive angiogenesis, and even releasing BMPs [15-18].

Once the bacteria (i.e., the antigenic stimulus) and cellular debris have been removed, M1 macrophages transform to an M2 variant also known as an alternatively activated macrophage such that the reparative process can begin. Indeed, the M2 variant is often referred to as a reparative macrophage to underscore its prominent role in restoring normal organ homeostasis as in fracture-related bone morphogenesis. The exact stimulus that causes the M1 (phagocytic) macrophage to convert to the M2 (reparative) macrophage is unknown but presumably occurs when the stimulus provided by the antigen load or the components of cellular debris falls below a certain level. M2 macrophages then release cytokines to attract pro-osteoblasts and mesenchymal stem cells to the fracture or surgical site to initiate bone healing [19].

The osteoconductive component of a graft comes into play here because the cells require boundaries and migration routes provided by a scaffold within which they will reside and operate. Induction is not required, since the bone macrophage’s preparatory pathway was already induced through attraction and stimulation of M0 monocytes and local tissue macrophages that follow antigenic chemical gradients, cell detritus, or cytokine signals to the site. This study demonstrates that involving the immune system early obviates the need for inducers such as nanotechnology or debris-mimicking peptides that require cellular contact to “induce” reparative stimulation or transformation.

Grafting materials largely require the requisite calcium products because calcium alone comprises about 40% of bone by weight and is arguably the most distinguishing element of the skeleton as a unique tissue. It is also highly probable that calcium ions or calcium-containing molecular substrates signal to reparative macrophages and resident stem cells that the tissue to be repaired is indeed bone [20]. Although synthetic graft materials may include stem cells or enhancing peptides and are frequently classed as osteoinductive (i.e., they actually stimulate bone formation), they vary widely in their ability to induce bone growth. Most are largely osteoconductive (i.e., they serve as a scaffold or conduit along which normal bone morphogenesis proceeds), and the efficacy of available products varies dramatically.

By antigenically coating the surface of an implant or graft with a potent immunologic inducer or enhancer of the immune system, an inert calcium-containing product alone can serve as an osteopreparatory, osteoinductive, and osteoconductive implant that fosters fully functional and robust bone fusion. Unlike surface nanotechnology, stem cell explants, or additive peptides mimicking traumatic cellular debris, there is no requirement that the macrophages randomly blunder and become activated by surface-to-surface contact; the presence of a potent antigen gradient chemotactically signals the macrophages to come locally and from the bloodstream to initiate repair. All cytokines, including various BMPs, are released at the appropriate times and in the required amounts. The presence of calcium ions or calcium-containing substrates indicates that the tissue to be repaired is bone [20].

**Figure 4:**
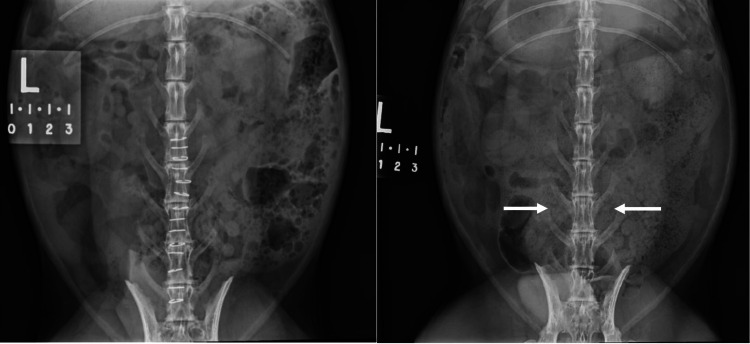
Anteroposterior radiographs obtained immediately postoperatively (left) and at 12 weeks (right). The arrows on the radiograph from 12 weeks’ follow-up demonstrate nonfusion and complete disappearance of the immunologically activated graft.

**Figure 5:**
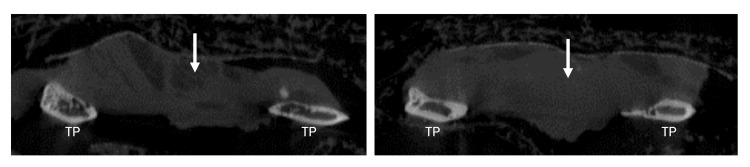
Sagittal micro-computed tomograms of a New Zealand white rabbit demonstrate nonfusion as well as complete disappearance of the immunologically activated graft at 12 weeks (arrows). TP: transverse process

In this study, in the rabbits that received the immunologically activated graft but did not fuse, the graft disappeared completely (Figures 4 - 5). Most cases of fusion nonunion have a modicum of residual bone and fibrous tissue [10]. The disappearance of the graft in these rabbits suggests that some grafts may have received a greater saturation of antigen than others, such that the M1 macrophages perceived all of the graft to be “contaminated” in those cases and, therefore, marked it for removal. If all of the graft is removed, there is no remaining conduit or scaffold on which to formulate bone.

The presumption of immune system activation was based on the addition of bacterial antigens to pure and otherwise unaltered electrospun beta-tricalcium phosphate. It is probable that simply controlling the concentration of antigen and/or relegating it to the periphery of the implant graft or scaffold would prevent the graft from being entirely consumed. Though this theory needs more study, it is possible that once the antigen is removed from the outer peripheral layers, the transition from M1 phagocytic macrophage to M2 reparative macrophage will occur while there is sufficient graft material left to allow the requisite cellular actors to formulate a bony fusion. The robust architecture of the antigenic implants and their superior performance on mechanical testing suggest that moderation of the dose and/or site of antigen application to a graft may well result in a less expensive bone grafting technique that could meet or exceed the efficacy of the gold standard autograft-all while avoiding the problems associated with harvesting autograft bone.

This study was limited by its small sample size and the comparison to historic controls; however, even in this limited experiment, the effects of antigenic stimulation on bone morphogenesis in a difficult animal fusion model (i.e., non-instrumented posterolateral fusion) were remarkable.

## Conclusions

Future directions regarding the incorporation of osteoimmunologic principles into bone healing are bright, and additional studies are clearly warranted to understand whether these options could improve upon our ability to facilitate superior bone arthrodesis in all orthopedic arenas, perhaps reducing costly surgical non-unions.

This pilot study merely hints at the untapped potential of the immune system in aiding bone healing and suggests that stimulating the bone morphogenetic pathway near the beginning of the process may well obviate the need for stem cells, nano surface characteristics, genetically engineered proteins, and pharmacologic doses of BMP or other cytokines, as the body best knows how to orchestrate this function after 400 million years of practice.
